# Successful use of intrapelvic Quikclot in life-threatening blast injury^☆^

**DOI:** 10.1016/j.tcr.2017.10.021

**Published:** 2017-11-08

**Authors:** Naveen Virin Goddard, Demetrius Evriviades

**Affiliations:** a College of Medical and Dental Sciences, University of Birmingham, Edgbaston, Birmingham B15 2TT, United Kingdom; b Queen Elizabeth Hospital Birmingham, Mindelsohn Way, Birmingham, B15 2TH, United Kingdom

**Keywords:** Quikclot, Intracorporeal, Intracavity, Blast, Trauma, Haemorrhage

## Abstract

Patients that suffer multiple traumatic injuries often present with uncontrollable haemorrhage and rapidly descend into a viscous death triad consisting of hypothermia, coagulopathy and acidosis. Initial surgical intervention does not aim to provide conclusive repair, but instead strives to stop blood loss while priority is given to correct the patient's metabolic state (Duchesne et al., 2010). However in some cases of massive polytrauma, gaining surgical control of bleeding can be incredibly difficult. As a result a number of topical haemostatic agents were developed for use in military and civilian settings. This case details a successful intracavity use of the granular haemostatic agent, Quikclot™ (Z-Medica), in halting massive haemorrhage in a patient who sustained major blast injuries. Although not officially recommended, intracorporeal uses of Quikclot™ can be effective as a last resort in preventing loss of life in cases of severe polytrauma. However, users need to remain wary of complications which may arise due to its application.

## Case report

**Fig. 1:**
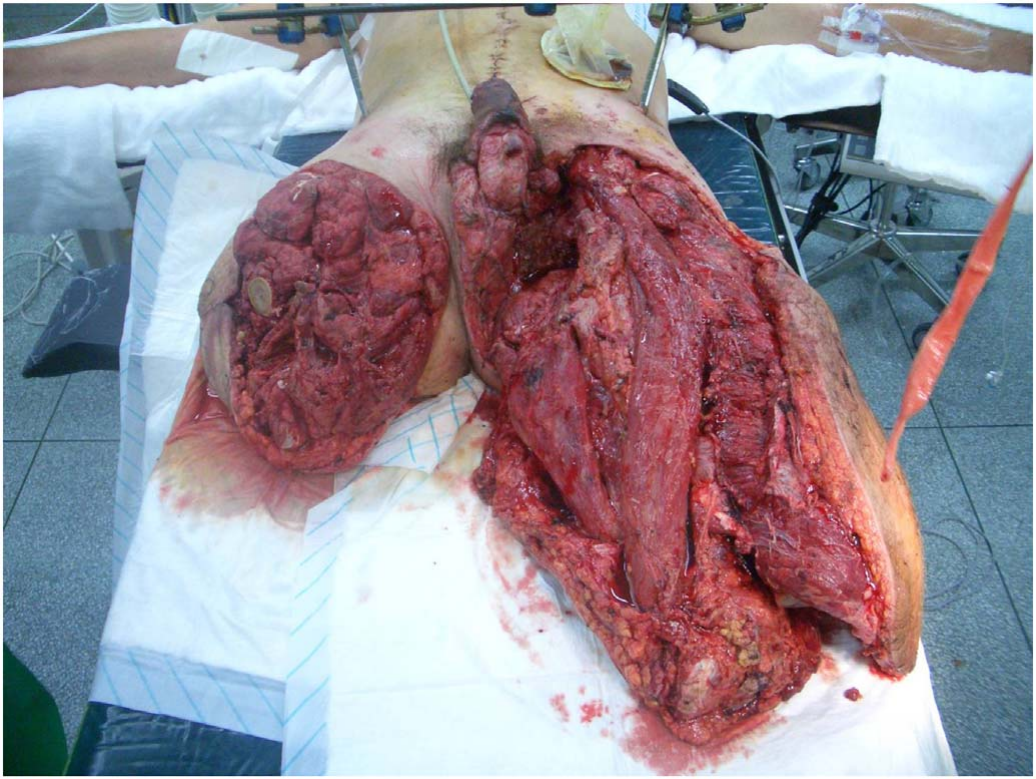
The extent of the injuries sustained by the patient.

A 28-year-old male sustained severe trauma when the vehicle he was driving was hit by an Improvised Explosive Device. The resulting injuries included a right traumatic above-knee amputation, left traumatic below-knee amputation, bilateral traumatic orchidectomy, an open pelvic fracture and massive soft tissue damage to the groin and perineum (Fig. 1). On assessment the patient scored 66 on the Injury Severity Scale.

**Fig. 2:**
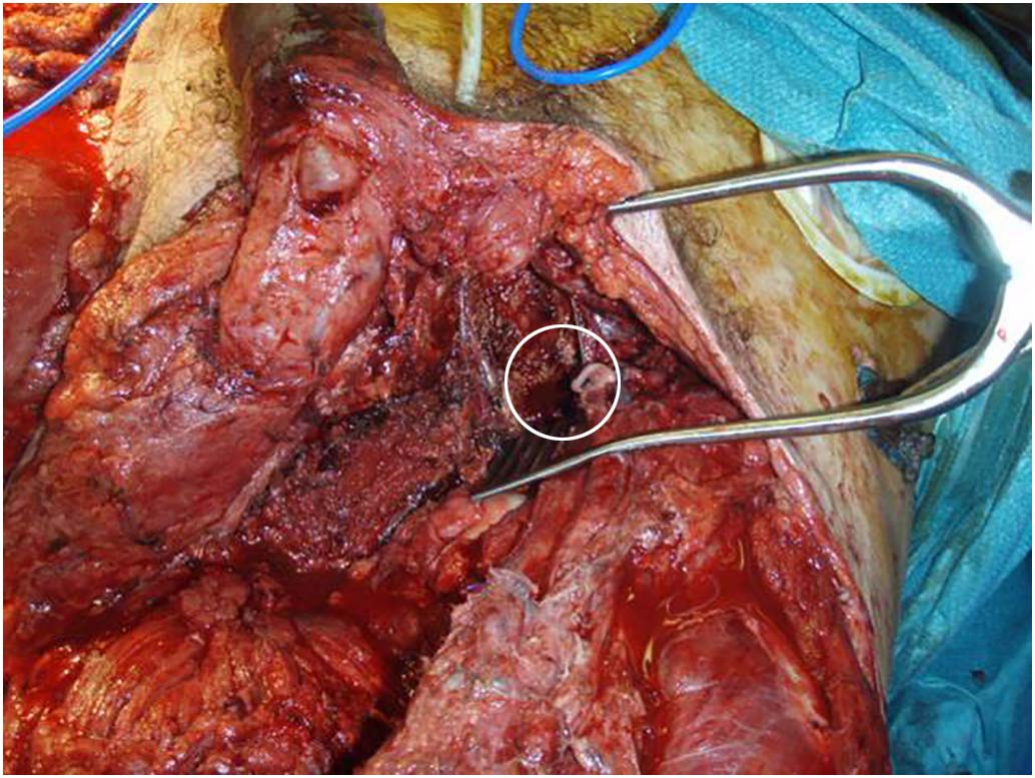
The pelvic cavity to which Quikclot granules were applied has been highlighted. The figure also shows the need for urethral reconstruction, thigh resurfacing and covering of the femoral vessels.

Immediately after the explosion bilateral combat tourniquets were applied to the patient. The loss of blood was so serious that Quikclot, a granular haemostatic device, was poured on the pelvic cavity to stem any further haemorrhage (Fig. 2). The patient was immediately intubated and underwent hypotensive fluid resuscitation as he was evacuated to Camp Bastion.

On primary survey the patient's airway was clear, normal bilateral breathing sounds were heard and despite being hypotensive both radial and right femoral pulses were present. The patient was persistently tachycardic (160 bpm), acidotic (pH = 4.87), hypoxic (pp0_2_ = 4.9 kPa) and had elevated lactate levels. A neurological assessment yielded a GCS score of five. The patient was was immediately transferred into theatre in a moribund state, displaying features of haemorrhagic shock.

An exploratory laparotomy was performed, the bowel was divided and thoroughly washed out. Ligation of the internal iliac and femoral veins was undertaken, this was not successful in stopping presacral bleeding despite the area being heavily packed. Elsewhere two large pads were used to stem abdominal bleeding, a further three swabs and two surgical sponges were used on the left groin wound. However the patient continued to be haemodynamically unstable and hypotensive.

Imaging of the pelvis revealed a vertical shear pelvic fracture with significant bleeding from the retroperitoneum and sacral ala. The pelvis was stabilized via external fixation and screwing of the sacroiliac joint.

At this point the patient's circulation was still volume demanding, with blood oozing continuously from the groin trauma sustained. The application of Quikclot, alongside a significant number of large pads and sponges, had stopped acute blood loss from the pelvic cavity and groin trauma. However, one day after the incident sanguineous fluid continued to seep from the perineum. This characteristic oozing eventually subsided and the patient's circulation stabilized. In total the patient received 55 units of packed red blood cells, 54 units of fresh frozen plasma, nine units of cryoprecipitate, seven units of platelets and 10 mg of rFVIIa.

The patient was intubated and ventilated for a total of 10 days. One week after the initial incident the left stump and vastus lateralis were debrided, the posterior compartment was thoroughly washed out and the external fixator removed. Further minor complications were promptly resolved and posed no serious threat to the patient.

**Fig. 3:**
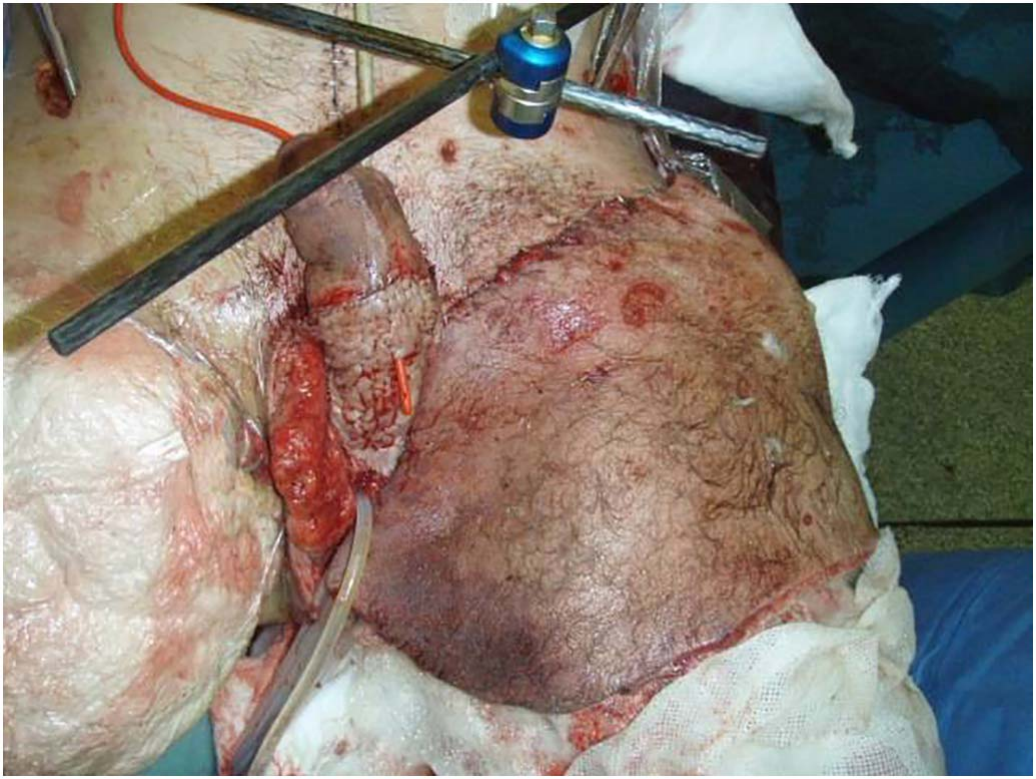
The results of a partial split skin graft to the left AKA and the penis.

**Fig. 4:**
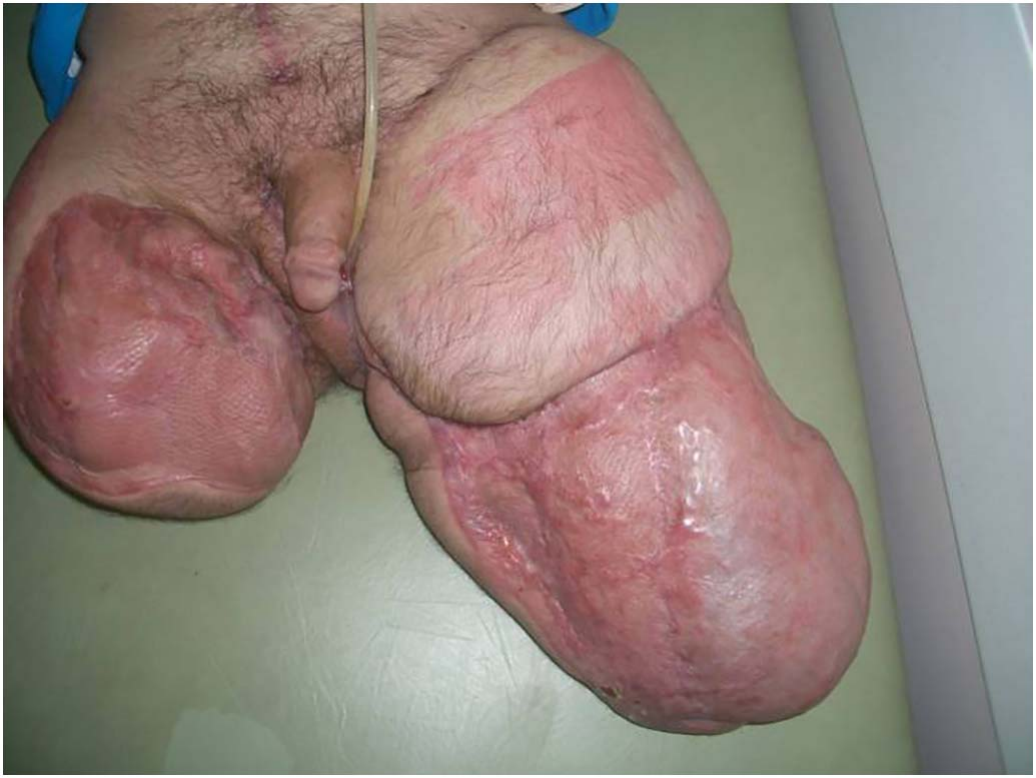
The results of the skin grafts and elective reconstructive surgery the patient underwent in the months after the blast.

The extent of the trauma sustained made extensive reconstruction crucial in this case. Split skin grafts were used to resurface the left AKA and the penis, a tensor fascia lata flap was used to fill the pelvic defect and cover the femoral vessels (Fig. 3). Further plastic reconstruction was carried out at the Royal Centre for Defence Medicine at Queen Elizabeth Hospital in Birmingham (Fig. 4). After a uneventful postoperative period the patient was discharged and has not been readmitted since.

## Discussion

Quikclot is an antihaemorrhagic agent that arrests significant blood loss. The first generation of the product consisted of zeolite beads that were applied directly onto injury sites to stop bleeding. The hydrophilic nature of the granules allowed water to be rapidly absorbed from the serum, increasing the concentration of coagulation cascade factors and platelets.

The efficacy of Quikclot in achieving haemostasis after vessel transection has been widely documented previously [1], [2], [3]. However the exothermic nature of the reaction has been known to cause split and partial thickness burns, which on one occasion required split-thickness grafting [4]. Further serious complications caused by Quikclot application have also been reported [5]. Therefore, as of January 2017 Quikclot products are still only recommended by the US military for compressible external haemorrhages which are not amenable to tourniquets [6].

Even with this recommendation, there have previously been case reports that have documented the successful use of Quikclot in intracorporeal injuries [7]. Ineffective examples have been attributed to the difficulty of direct application onto inaccessible injuries and the diluted blood volumes and coagulopathy of patients. These both reduce the impact of the hydrophilic absorption promoted by Quikclot [8]. In this particular case, despite the patient's haemorrhage and deteriorating metabolic state, intracavity Quikclot was still successful in stopping blood loss after all other attempts had failed.

Remarkably in this instance, aside from the initial debridement in the hours after the blast, debridement of the lateral thigh, perineum, groin and amputated stumps were carried out six times in total. However this level of contamination is not unusual in cases of blast injury, therefore further contamination by granules was seen to be of little consequence as wounds would have been debrided, copiously irrigated and left open anyway. The main threat posed in this case was a possible foreign body reaction and infection - neither of which transpired.

This case is set apart from previous examples by the almost unparalleled injuries sustained by the soldier involved. With a ISS score of 66 he was one of the most severely injured surviving combat personnel in the conflict in Afghanistan, and despite his extensive injuries Quikclot application was successful in stemming bleeding from his pelvic cavity. This is testament to the ease of application and efficacy of the agent in dealing with haemorrhage as a result of polytrauma. Any additional complications associated with Quikclot use are vastly outweighed by the threat of a patient dying due to excessive exsanguination.

To conclude, this report chronicles a case of intracavity Quikclot use which was successful in saving the life of a soldier who suffered almost unprecedented levels of trauma due to an IED. The application of granular Quikclot in this case allowed the patient, despite his injuries, to be one of the most remarkable unexpected survivors from the war in Afghanistan. However, the current generation of this product is still only recommended for external injuries. Any intracorporeal use of Quikclot should only be completed to prevent the loss of life, and even in those extreme circumstances consideration needs to be given to possible complications which may arise as a result of its application.

## References

[1] Alam H.B., Uy G.B., Miller D., Koustova E., Hancock T., Inocencio R. (2003). Comparative analysis of hemostatic agents in a swine model of lethal extremity injury. J. Trauma.

[2] Alam H.B., Chen Z., Jaskille A., Querol R.I., Koustova E., Inocencio R. (2004). Application of a zeolite hemostatic agent achieves 100% survival in a lethal model of complex groin injury in swine. J. Trauma.

[3] Pusateri A.E., Delgado A.V., Dick E.J., Martinez R.S., Holcomb J.B., Ryan K.L. (2004). Application of a granular mineral-based hemostatic agent (Quickclot) to reduce blood loss after grade V liver injury in swine. J. Trauma.

[4] Wright J.K., Kalns J., Wolf E.A., Traweek F., Schwarz S., Loeffler C.K. (2004). Thermal injury resulting from application of a granular mineral hemostatic agent. J. Trauma.

[5] Plurad D., Chandrasoma S., Best C., Rhee P. (2009). A complication of Intracorporeal use of Quikclot for pelvic hemorrhage. J. Trauma.

[6] The Committee on Tactical Combat Casualty Care TCCC Guidelines for Medical Personnel. http://cotccc.com/wp-content/uploads/TCCC-Guidelines-for-Medical-Personnel-170131.pdf.

[7] Wright F.L., Hua H.T., Velmahos G., Thoman D., Demitriades D., Rhee P.M. (2004). Intracorporal use of the hemostatic agent QuickClot in a coagulopathic patient with combined thoracoabdominal penetrating trauma. J. Trauma.

[8] Rhee P., Brown C., Martin M., Salim A., Plurad D., Green D. (2008). QuikClot use in trauma for hemorrhage control: case series of 103 documented uses. J. Trauma.

