# Corrigendum to “Successful use of intrapelvic Quikclot in life-threatening blast injury” [Trauma Case Rep. 12 (2017) 59–62]

**DOI:** 10.1016/j.tcr.2018.03.001

**Published:** 2018-03-20

**Authors:** Naveen Virin Goddard, Demetrius Evriviades

**Affiliations:** a College of Medical and Dental Sciences, University of Birmingham, Edgbaston, Birmingham B15 2TT, United Kingdom; b Queen Elizabeth Hospital Birmingham, Mindelsohn Way, Birmingham B15 2TH, United Kingdom

The author regrets that the second author, Demetrius Evriviades, was inadvertently missed from the author list. The author list has now been updated with the second author.

The authors would like to apologise for any inconvenience caused.

